# County Smoke-Free Laws and Asthma Discharges: Evidence from 17 US States

**DOI:** 10.1155/2017/6321258

**Published:** 2017-05-14

**Authors:** Glenn M. Landers, Patricia Ketsche, Mark L. Diana, Claudia Campbell

**Affiliations:** ^1^Georgia Health Policy Center, Georgia State University, 55 Park Place, 8th Floor, Atlanta, GA 30303, USA; ^2^Robinson College of Business, Georgia State University, 35 Broad St., Atlanta, GA 30303, USA; ^3^Department of Global Health Systems Development, Tulane University School of Public Health and Tropical Medicine, New Orleans, LA, USA

## Abstract

**Background:**

Although approximately 82 percent of the US population was covered by some form of law that restricted smoking in public establishments as of 2014, most research examining the relationship between smoke-free laws and health has been focused at the state level.

**Purpose:**

To examine the effect of county workplace smoke-free laws over and above the effect of other (restaurant or bar) smoke-free laws on adult asthma.

**Methods:**

The study estimated the effect of rates of adult asthma discharges before and after the implementation of county nonhospitality workplace smoke-free laws and county restaurant and bar smoke-free laws. Data were from 2002 to 2009, and all analyses were performed in 2011 through 2013.

**Results:**

A statistically significant relationship (−5.43, *p* < .05) was found between county restaurant or bar smoke-free laws and reductions in working age adult asthma discharges. There was no statistically significant effect of nonhospitality workplace smoke-free laws over and above the effect of county restaurant or bar laws.

**Conclusions:**

This study suggests that further gains in preventable asthma-related hospitalizations in the US are more likely to be made by focusing on smoke-free laws in bars or restaurants rather than in nonhospitality workplaces.

## 1. Introduction

A 2006 Centers for Disease Control and Prevention (CDC) report estimated that more than 10 percent of all tobacco-related deaths were attributable to exposure to secondhand smoke (SHS), and the same report documented the effects of exposure to SHS on the exacerbation of respiratory diseases such as asthma [1]. While the CDC deemed the evidence sufficient for the existence of a causal relationship between exposure to SHS and increased acute respiratory illnesses and related hospital discharges among children, this finding was only suggestive for adults. A 2014 report update estimated that, over the past 50 years, 2.5 million deaths have been among nonsmokers who died from diseases caused by exposure to SHS [2].

Multiple strategies have been implemented to lower the number of Americans who smoke and to protect nonsmokers from the effects of SHS. Policy interventions in the private sector have ranged from surcharges on employer-sponsored and commercial health insurance policies for those who smoke [3, 4] to bans on smoking in the workplace [5, 6]. Public sector interventions include restrictions on tobacco advertising in the media and at the point of sale [7, 8], cigarette taxes targeted at raising the explicit cost of smoking [9, 10], and the implementation of smoke-free laws at either the state or local (city or county) levels [6, 11, 12].

According to the American Nonsmokers' Rights Foundation (ANRF), as of July, 2014, 1,135 US municipalities (cities or counties) had laws in effect that required 100 percent smoke-free (nonhospitality) workplaces, bars, restaurants, or combinations thereof [13]. ANRF defines 100 percent smoke-free restaurants and bars as banning all smoking without exception. With respect to workplaces, minor exceptions may be made for sole proprietorships, family-owned businesses where all employees are related to the owner, and jails [13]. Although approximately 82 percent of the US population was covered by some form of state, county, or local (city) law that restricted smoking in some type of venue as of 2014, most of the research examining the relationship between smoke-free laws and health outcomes has focused on state laws [14, 15].

Further, the workplace is one venue in which the private sector may have been ahead of state and local governments in instituting smoking restrictions. Throughout the 1990s, nonhospitality workplaces increasingly adopted no-smoking policies [16, 17], and by the early 2000s, almost 70 percent of nonhospitality workplaces were covered by smoking bans [18]. It is fair to ask, then, if further expansion of nonhospitality workplace smoke-free laws provides any added health benefit or if resources would be better allocated pursuing laws that cover restaurants and bars.

In 2008, Rayens et al. studied Lexington-Fayette County, Kentucky emergency department (ED) visits for asthma in four of five area hospitals before and after the implementation of a county smoke-free law (2001–2006) covering all public places but not all places of employment [19]. ED asthma visits declined 22 percent from prelaw to postlaw, and the decline was greater for adults than for children. In 2010, Naiman and colleagues studied the effect of the Toronto smoke-free law three years before the first phase of implementation and two years after the law was fully implemented [20]. The Toronto law was phased in from 1999 to 2001 and covered 2.5 million residents. The first phase required all public places and workplaces to be smoke-free; the second phase required all restaurants, dinner theatres, and bowling centers, except for designated smoking rooms, to be smoke-free; the third and final stage required all bars, billiard halls, bingo halls, casinos, and racetracks, except for designated rooms, to be smoke-free. There was no significant reduction in asthma discharges after the implementation of the law's first phase affecting public spaces and workplaces. Discharges for respiratory conditions fell 33 percent over three years after the restaurant law went into effect. There was no significant reduction in asthma discharges after the third phase was implemented affecting bars and pool halls.

Shetty et al. [6] examined changes in hospitalization and mortality rates for various causes, including asthma, following the implementation of smoke-free laws using the HCUP Nationwide Inpatient Sample (NIS) data for 1993 to 2004, Medicare claims for 1997 to 2004, and the Multiple Cause of Death database for 1989 to 2004. The authors performed a hospital-level analysis and matched hospitals in areas with smoke-free laws to hospitals in areas without smoke-free laws. They found significant reductions in asthma admissions for working age adults related to workplace laws that disappeared after adjusting for multiple comparisons. In a study of Olmstead County, MN, Hurt et al. found no significant reduction in heart attacks after a restaurant smoke-free law was implemented but found a 35 percent decline in heart attack after a law was implemented five years later covering all workplaces, including bars [11].

Thus, the literature is inconclusive about the impact of different types of US county smoke-free laws on adult health outcomes, particularly for asthma. We hypothesize that the effectiveness of nonhospitality workplace smoke-free laws will depend on whether businesses in the county have already implemented smoking restrictions in the workplace, as suggested by the literature, and whether the laws are in place for other establishments such as bars and restaurants. At this point in time, the implementation of county smoke-free laws in other venues may be relatively more effective than implementing smoke-free laws in workplaces that exclude bars and restaurants.

This study is the first multistate, county level analysis using Healthcare Cost and Utilization Project (HCUP) state inpatient data (SID) to examine the effect of county nonhospitality workplace smoke-free laws over and above the effect of other (restaurant or bar) smoke-free laws on adult asthma. It adds to the literature by building upon the evidence for the effect of smoke-free laws on adult asthma, by focusing the analysis at the county rather than the state level, and by analyzing the relative effects of the implementation of 100% smoke-free nonhospitality workplace laws versus 100% smoke-free bar and restaurant laws on asthma.

## 2. Methods

The study employed a pre/post, nonequivalent control group design using ordinary least squares (OLS), fixed effects models to estimate the change in quarterly county rates of adult asthma discharges before and after the implementation of 100% smoke-free nonhospitality workplace laws in counties where no law or a less than 100% smoke-free law (a qualified law) was previously in force as compared with the implementation of 100% smoke-free restaurant and bar laws where no laws or qualified laws had previously been implemented. All analyses were performed in 2011 through 2013 with data from 2002 to 2009.

### 2.1. Study Data and Sample Selection

Study data were drawn from two main sources: the HCUP SID from 2002 to 2009 and the April, 2011, ANRF smoke-free laws database of state and county laws. Therefore, any law passed by a state or county after that date is not included in this study. Counties were classified as having a smoke-free law only if the county law was categorized as 100% smoke-free. A county nonhospitality workplace law is defined by ANRF as 100% smoke-free as follows:

  All workplaces must be completely smoke-free, with some minor exceptions: (A) workplaces with only one employee are exempt. (B) Family-owned businesses and businesses run by self-employed persons, in which all the employees are related to the owner or the self-employed person and which are not open to the public are exempt. (C) With respect to public workplaces, jails or interrogation rooms are exempt [21].

A county bar or restaurant law is considered 100% smoke-free as follows:

  All restaurants, including attached bars, must be completely smoke-free, without exception. All freestanding bars must be completely smoke-free, without exception [21].

Where a 100% smoke-free municipal law was present without a corresponding county law, the county was labeled as having a 100% smoke-free law, as that was seen as the more conservative approach in terms of interpreting its impact on the majority of the population in the county. (From this point forward, 100% smoke-free laws are simply referred to as smoke-free laws.)

The initial selection of counties for inclusion in this study was based on data derived from an earlier study of the effects of state smoke-free laws [22], so inclusion was dependent on the date each state implemented a smoke-free law and the availability of each state's HCUP data. Of the 35 states that had some type of smoke-free law as of April, 2011, thirty-two participated in HCUP, and 23 participated in HCUP's standardized data program. Nine of these states' smoke-free laws were implemented too far in the past or too recently to enable comparison with other states and counties, as there were no HCUP data available for those dates. Further, Massachusetts and Nevada did not report patient discharges by county of residence, so those states' data were omitted as well. Counties in the remaining 12 states were included in the study sample as treatment counties.

Six other states participated in the HCUP standardized data program but had not enacted a smoke-free state law by April, 2011, or data were unavailable to analyze the law's postimplementation period. West Virginia does not report patient data by county of residence, so its data were omitted. The counties in these remaining five states were included in the sample as control counties.

### 2.2. Model Specification

The dependent variable (rate_*ct*_) was the quarterly county rate of asthma discharges per 10,000 working age adults in county *c* at time *t*, where patient residence defined county. The independent variables included a dummy variable (work_*ct*_) indicating the presence (1) or absence (0) of a county smoke-free nonhospitality workplace law in county *c* at time *t*. A second dummy variable (other_*ct*_) indicated the presence (1) or absence (0) of a county smoke-free restaurant or bar law in county *c* at time *t*. An interaction term (work_*ct*_*∗*other_*ct*_) tested the interaction of a county having a nonhospitality workplace law and a restaurant or bar law in county *c* at time *t*.

A cigarette tax variable from Tax Foundation data was included to adjust for state cigarette taxes, as such taxes might contribute to increases or decreases in smoking and the prevalence of SHS. A year variable controlled for underlying time effects unrelated to smoke-free law implementation. A state law dummy variable controlled for whether or not there was a state 100% smoke-free law of any type in effect before or after the county law, as the presence of state laws might moderate the effect of county laws. A seasonal variable (winter, spring, summer, and fall) controlled for seasonal allergies that might otherwise exacerbate asthma discharges. Additional, observable county characteristics that may also impact asthma discharges were included from Behavioral Risk Factor Surveillance System, HCUP SID, Small Area Poverty Estimates, Small Area Health Insurance Estimates, Area Resource File, and US Census Bureau data, including smoking and asthma prevalence, rurality, percent of population in poverty, the percentage of uninsured, the number of primary care providers, hospitals, and teaching hospitals per 10,000, residents, the percentage of residents who smoked, and county racial composition.

The model included age-adjusted population weights to account for variations in county population that might skew discharge rates [23, 24]. The Stata* robust cluster* option was used to adjust for potential serial correlation and heteroskedasticity [25, 26]. Overall model fit was assessed using Fisher's* F*-test. Statistical significance was measured using Student's* t*-test at the .05 level. A separate model was run using quarterly appendicitis discharge rates as the dependent variable to test relative changes in discharges in the same counties and over the same time frame for a condition unrelated to SHS exposure as indicated by Hill as a test of plausibility [27]. Institutional Review Board approval was granted from Tulane University and Georgia State University. All analyses were performed using Stata statistical software version 11.

## 3. Results

The combined population of counties in the 17 states included in the study was more than 103,000,000 individuals or about 35 percent of the US population, in 2005. In total, 29 counties out of 840 counties included in the study passed county clean indoor air laws during the three years for which each state's data were analyzed, 16 counties already had a county nonhospitality workplace law in the first observed quarter, 20 already had a county restaurant or bar law in the first observed quarter, and 785 had no county smoke-free law during the three years. Additionally, 444 counties were in states that passed state smoke-free laws over the three years.

Descriptive statistics are presented in Table 1 and were comparable to national rates and averages. The mean adult asthma discharge rate per 10,000 was almost 14 per quarter. County rates ranged as high as 89 per 10,000. The appendicitis discharge rate ranged up to 182 per 10,000 per quarter in one county. The average cigarette tax was 82 cents per pack. On average, 22 percent of county residents smoked in the study years, and just over eight percent had asthma. The percent of county population living in poverty and the percent uninsured ranged widely. Some counties had no primary care physician, but the mean was just over six per 10,000. Some counties had no racial diversity, while one county had a nonwhite percentage of 86 percent.

Bivariate results are presented in Table 2. Asthma discharge rates were positively associated with being nonwhite (.26, *p* < .001), living in poverty (.19, *p* < .001), and having more primary care physicians per capita in the county of residence (.16, *p* < .001). All of these variables were included as control variables in the multivariate models.

Multivariate results are presented in Table 3. A statistically significant relationship (−5.43, 95% CI = −10.5, −.4) was found between the implementation of county restaurant or bar smoke-free laws and reductions in working age adult asthma discharges. However, there was no statistically significant effect of the passage of smoke-free nonhospitality workplace laws on working age adult asthma discharges over and above the effect of county restaurant or bar laws. In the appendicitis model, there was no statistically significant relationship between the implementation of county smoke-free laws and appendicitis discharges.

## 4. Discussion

Our results indicate that asthma discharges were not reduced by enacting smoke-free workplace laws within county jurisdictions. There does appear to be a relationship between enacting smoke-free restaurant and bar laws and reductions in asthma discharges, however. This may indicate agreement with the literature that shows the private sector has been successful in voluntarily reducing SHS exposure in the workplace through workplace smoke-free policies. For example, Brownson et al. report that, by 1999, 79 percent of US workplaces were covered by some kind of formal no-smoking policy [16], and Farrelly et al. showed that, even earlier in the 1990s, 82 percent of US workers were covered by some type of smoking restriction [17]. Our results indicate that additional restrictions brought about by enacting county workplace laws may have limited effectiveness on asthma discharges. Instead, implementing smoke-free bar and restaurant laws would be more effective in further reducing SHS and associated asthma discharges among adults.

Overall, our findings suggest that further reducing SHS exposure among adults who work in or patronize bars and restaurants that allow smoking does reduce asthma-related discharges and, therefore, such smoking bans have the potential to improve health. Our study only looked at the implementation of 100% smoke-free laws in nonhospitality workplaces, bars, and restaurants. Implementing less restrictive smoke-free (nonhospitality) workplace laws where no county law existed or where employers have not voluntarily implemented private policies may still have the potential to reduce asthma discharges, but less so than implementing bar and restaurant laws. This was not addressed and warrants further research.

The major limitation of our study is the potential bias introduced by the selection of study states from which county data were drawn. We removed states from the analysis where needed data were not available either because the state did not participate in HCUP, the timing of their participation in HCUP was too recent, or the timing of the passage of smoke-free laws in the state was too recent or long ago. To the extent that omitted factors related to variation in county asthma discharges are also related to the exclusion of states (and their counties) from our sample, our results may be biased.

Only 49% of the US population lives in a location where smoke-free laws are in force in all bars, restaurants, and workplaces [28]. As of April 2011, 2,919 (93%) of 3,144 US counties (or county equivalents in the ANRF database) had no workplace 100% smoke-free laws in force, while 2,996 (95%) had no 100% smoke-free laws in bars and 2,918 (or 93%) had no 100% smoke-free laws in restaurants. Clearly, opportunities to further reduce SHS exist through changes in county smoking laws.

This study suggests that further gains in preventable asthma-related illness in the US are more likely to be made by focusing on antismoking laws in bars or restaurants rather than nonhospitality workplaces. Assuming these results can be generalized to all US counties in our study that did not have bar or restaurant smoke-free laws in place, we estimate that an additional 467 asthma-related hospital discharges could have been averted per year.

## Figures and Tables

**Table 1:** Descriptive statistics for continuous study variables (county level data).

Variable	Mean	Standard deviation	Minimum	Maximum
*Dependent variables*				
Adult asthma rate	13.95	7.52	0	88.93
Adult appendicitis rate	2.26	2.45	0	181.82
*Independent variables*				
Cigarette tax	.82	.63	.03	2.58
Smoking prevalence	21.84	4.61	9.80	32.60
Asthma prevalence	8.24	1.73	6.10	40.50
Percent in poverty	13.70	5.73	0	43.80
Percent uninsured	15.73	4.96	5.48	39.50
PCP per 10,000	6.33	4.15	0	31.40
Percent nonwhite	11.00	14.00	0	86.00

**Table 2:** Factors associated with asthma discharge rates per 10,000.

Variable	Pearson's *ρ*	*p* value
Cigarette tax	.09	<.001
Smoking prevalence	.05	<.001
Asthma prevalence	.07	<.001
Percent in poverty	.19	<.001
Percent uninsured	−.08	<.001
PCP per 10,000	.16	<.001
Percent nonwhite	.26	<.001

**Table 3:** Effects of changes in county smoke-free laws on hospital discharges for asthma among working age adults.

	Working age adult asthma	Working age adult appendicitis
	Coefficient (RSE)	Coefficient (RSE)

Passage of a county 100% smoke-free workplace law	−5.94 (3.89)	0.26 (0.20)
Passage of a county 100% smoke-free restaurant or bar law	−5.43^*∗*^ (2.56)	0.05 (0.27)
County workplace smoke-free law^*∗*^ county restaurant or bar smoke-free law	11.88 (7.10)	−0.16 (0.38)
Cigarette tax	3.15^*∗∗*^ (0.54)	0.38^*∗∗*^ (0.06)
Smoking prevalence	−0.04 (0.16)	−0.03^*∗*^ (0.01)
Asthma prevalence	0.16^*∗∗*^ (0.04)	−0.01^*∗*^ (0.00)
Percent in poverty	0.68^*∗∗*^ (0.16)	−0.02 (0.01)
Percent uninsured	−0.13 (0.10)	0.03^*∗∗*^ (0.01)
Presence of any state smoke-free law	−0.38 (0.68)	−.06 (0.05)
Constant	5.62 (2.98)	2.18^*∗∗*^ (0.44)

RSE: robust standard error; ^*∗*^*p* < .05; ^*∗∗*^*p* < .01.

## References

[1] CDC (2006). The health consequences of involuntary exposure to tobacco smoke: a report of the Surgeon General. *Services USDoHaH*.

[2] CDC (2014). The health consequences of smoking: 50 years of progress. a report of the surgeon general. *Centers for Disease Control and Prevention NCfCDPaHP, Office on Smoking and Health*.

[3] Penner M. (1989). Economic incentives to reduce employee smoking: a health insurance surcharge for tobacco using State of Kansas employees. *American Journal of Health Promotion*.

[4] Lecker M. J. (2009). The smoking penalty: distributive justice or smokism?. *Journal of Business Ethics*.

[5] Eriksen M. P., Cerak R. L. (2008). The diffusion and impact of clean indoor air laws. *Annual Review of Public Health*.

[6] Shetty K. D., DeLeire T., White C., Bhattacharya J. (2011). Changes in U.S. hospitalization and mortality rates following smoking bans. *Journal of Policy Analysis and Management*.

[7] Frick R. G., Klein E. G., Ferketich A. K., Wewers M. E. (2012). Tobacco advertising and sales practices in licensed retail outlets after the food and drug administration regulations. *Journal of Community Health*.

[8] Luke D. A., Ribisl K. M., Smith C., Sorg A. A. (2011). Family smoking prevention and tobacco control act: banning outdoor tobacco advertising near schools and playgrounds. *American Journal of Preventive Medicine*.

[9] Callison K., Kaestner R. (2014). Do higher tobacco taxes reduce adult smoking? new evidence of the effect of recent cigarette tax increases on adult smoking. *Economic Inquiry*.

[10] Carpenter C., Cook P. J. (2008). Cigarette taxes and youth smoking: New evidence from national, state, and local Youth Risk Behavior Surveys. *Journal of Health Economics*.

[11] Hurt R. D., Weston S. A., Ebbert J. O. (2012). Myocardial infarction and sudden cardiac death in Olmsted County, Minnesota, before and after smoke-free workplace laws. *Archives of Internal Medicine*.

[12] CDC (2010). *Reducing Exposure to Environmental Tobacco Smoke. In: Services TFoCP, Guide to Community Preventive Services*.

[13] ANRF (2014). *Overview List—How many Smokefree Laws?*.

[14] Carpenter C. S. (2009). The effects of local workplace smoking laws on smoking restrictions and exposure to smoke at work. *Journal of Human Resources*.

[15] Gonzalez M., Sanders-Jackson A., Song A. V., Cheng K.-W., Glantz S. A. (2013). Strong smoke-free law coverage in the United States by race/ethnicity: 2000–2009. *American Journal of Public Health*.

[16] Brownson R. C., Hopkins D. P., Wakefield M. A. (2002). Effects of smoking restrictions in the workplace. *Annual Review of Public Health*.

[17] Farrelly M. C., Evans W. N., Sfekas A. E. S. (1999). The impact of workplace smoking bans: Results from a national survey. *Tobacco Control*.

[18] Levy D. T., Friend K. B. (2003). The effects of clean indoor air laws: What do we know and what do we need to know?. *Health Education Research*.

[19] Rayens M. K., Burkhart P. V., Zhang M. (2008). Reduction in asthma-related emergency department visits after implementation of a smoke-free law. *Journal of Allergy and Clinical Immunology*.

[20] Naiman A., Glazier R. H., Moineddin R. (2010). Association of anti-smoking legislation with rates of hospital admission for cardiovascular and respiratory conditions. *Canadian Medical Association Journal*.

[21] ANRF (2011). *Overview List—How many Smokefree Laws?*.

[22] Landers G. (2014). The impact of smoke-free laws on asthma discharges: a multistate analysis. *American Journal of Public Health*.

[23] Lott J. R., Whitley J. (2003). Measurement error in county-level UCR data. *Journal of Quantitative Criminology*.

[24] McLaughlin C. G., Normolle D. P., Wolfe R. A., McMahon L. F., Griffith J. R. (1989). Small-area variation in hospital discharge rates: do socioeconomic variables matter?. *Medical Care*.

[25] Bertrand M., Duflo E., Mullainathan S. (2004). How much should we trust differences-in-differences estimates?. *The Quarterly Journal of Economics*.

[26] StataCorp. (2009). *Stata: Release 11, Statistical Software*.

[27] Hill A. B. (1965). The environment and disease: association or causation?. *Proceedings of the Royal Society of Medicine*.

[28] ANRF (2014). *Summary of 100% Smokefree State Laws and Population Protected by 100% U.S. Smokefree Laws*.

